# POTION: an end-to-end pipeline for positive Darwinian selection detection in genome-scale data through phylogenetic comparison of protein-coding genes

**DOI:** 10.1186/s12864-015-1765-0

**Published:** 2015-08-01

**Authors:** Jorge A. Hongo, Giovanni M. de Castro, Leandro C. Cintra, Adhemar Zerlotini, Francisco P. Lobo

**Affiliations:** Laboratório Multiusuário de Bioinformática, Embrapa Informática Agropecuária, Empresa Brasileira de Pesquisa Agropecuária (Embrapa), Campinas, São Paulo 13083-886 Brazil

**Keywords:** Genome-scale positive selection detection, Comparative genomics, Molecular Darwinian positive selection

## Abstract

**Background:**

Detection of genes evolving under positive Darwinian evolution in genome-scale data is nowadays a prevailing strategy in comparative genomics studies to identify genes potentially involved in adaptation processes. Despite the large number of studies aiming to detect and contextualize such gene sets, there is virtually no software available to perform this task in a general, automatic, large-scale and reliable manner. This certainly occurs due to the computational challenges involved in this task, such as the appropriate modeling of data under analysis, the computation time to perform several of the required steps when dealing with genome-scale data and the highly error-prone nature of the sequence and alignment data structures needed for genome-wide positive selection detection.

**Results:**

We present POTION, an open source, modular and end-to-end software for genome-scale detection of positive Darwinian selection in groups of homologous coding sequences. Our software represents a key step towards genome-scale, automated detection of positive selection, from predicted coding sequences and their homology relationships to high-quality groups of positively selected genes. POTION reduces false positives through several sophisticated sequence and group filters based on numeric, phylogenetic, quality and conservation criteria to remove spurious data and through multiple hypothesis corrections, and considerably reduces computation time thanks to a parallelized design. Our software achieved a high classification performance when used to evaluate a curated dataset of *Trypanosoma brucei* paralogs previously surveyed for positive selection. When used to analyze predicted groups of homologous genes of 19 strains of *Mycobacterium tuberculosis* as a case study we demonstrated the filters implemented in POTION to remove sources of errors that commonly inflate errors in positive selection detection. A thorough literature review found no other software similar to POTION in terms of customization, scale and automation.

**Conclusion:**

To the best of our knowledge, POTION is the first tool to allow users to construct and check hypotheses regarding the occurrence of site-based evidence of positive selection in non-curated, genome-scale data within a feasible time frame and with no human intervention after initial configuration. POTION is available at http://www.lmb.cnptia.embrapa.br/share/POTION/.

**Electronic supplementary material:**

The online version of this article (doi:10.1186/s12864-015-1765-0) contains supplementary material, which is available to authorized users.

## Background

Maturation of second-generation sequencing technologies has created a wealth of genomic data to be systematically analyzed through several comparative genomic strategies in order to extract biological information from the patterns of conservation and variation observed in genomic elements shared within genomes [1–3]. A mainstream analysis in the field of comparative genomics is the genome-scale computational search for groups of homologous genes evolving under positive Darwinian selection, usually defined as genes with an elevated nonsynonymous substitution rate, since these groups of genes are of most interest to the understanding of how evolution works at the molecular level [4, 5].

Studies of this nature have been used to detect genes involved in speciation [6] and in the emergence of new phenotypic traits that increase evolutionary fitness [7–9]. Genome-scale searches for positive selection were also widely used to detect genes involved in host-pathogen co-evolutionary “arms race” in the genomes of several important pathogenic taxa such as *Escherichia coli* [10, 11], *Salmonella* [12], *Staphylococcus* [13], *Streptococcus* [14], *Trypanosoma brucei* [15] and *Campylobacter* [16], among many others. On the host side, a significantly high number of genes involved in immunity-related processes were also detected in genome-wide searches for positive selection in mammalian genomes [8].

While the considerable number of genome-scale positive selection detection (GSPSD) studies generated a substantial amount of valuable biological information, there is a lack of specialized software to perform such task in a general, automated, fast and statistically sound manner. Several factors are responsible for this scenario. One important aspect is the fact that the automatic detection of positive selection on molecular data is not trivial from the computational point of view, requiring the generation of data structures computationally costly to be calculated. It’s prohibitive to run analyses on thousands of groups of homologs, such as in multiple sequence alignment, phylogenetic tree reconstruction and fitting of distinct codon evolutionary models to the data, using single processor software within a feasible time frame [17].

Another important aspect is the highly error-prone nature of the sequence and alignment data structures needed for GSPSD [18]. Several sources of error that can generate spurious positive selection detection are produced during common bioinformatics procedures, such as in genome assembly and gene prediction. Among these errors are frame shifts, sequence ambiguities, gene fragments, chimeric sequences and pseudogenes considered as functional coding regions. Other common sources of error include the recruiting of excessively divergent sequences to groups of homologous genes during automatic homology prediction. All of the aforementioned errors can generate spurious alignment of non-homologous codons and significantly interfere with the reliable detection of positive selection [18, 19]. The occurrence of recombination events within homologous sequences can also significantly interfere with reliable GSPSD, since the codon evolution models commonly used to detect positive selection do not take into account recombination as a possible source of variation of homologous positions and assume all the columns of a multiple codon alignment to share the same evolutionary story [20]. Several predicted groups of homologous genes also contain mixed sets of 1-1 orthologs and paralogs, two biologically distinct gene groups that should be evaluated separately to investigate different biological questions [21]. Finally, the simultaneous search for recombination and/or positive selection in several groups of homologs creates a multiple hypothesis-testing scenario that requires correct statistical treatment to control the frequency of Type 1 errors [8, 22].

Here we report POTION (POsitive selecTION), a unique end-to-end modular, customizable and parallelized pipeline that overcomes the above stated challenges to detect positive selection on genome-scale data in batch mode. POTION allows users to easily and quickly survey their own genomic data of interest–large numbers of predicted genes and their homology relationships–for signs of positive selection. We demonstrate POTION is able to classify a curated dataset of *T. brucei* paralogs previously surveyed for positive selection with high accuracy. As a case study to illustrate some of the unique features found in POTION, such as the sophisticated sequence and groups filters and the heavily parallelized design, we applied our program to survey the complete set of coding sequences of 19 *Mycobacterium tuberculosis* strains using distinct configuration sets to specifically stress how such features dramatically change the number and the quality of groups of homologs predicted to evolve under positive selection, or the time to process genome-scale datasets. POTION detected several groups of positively selected homologous genes with known roles in the host-pathogen “arms race”, as expected for genes under Darwinian selection in a parasitic species. An extensive literature review found no single pipeline that contains all the software, features and flexibility tied together in an integrated environment to perform GSPSD in an automated manner. To researchers lacking bioinformatics expertise, POTION offers the first end-to-end workflow to perform GSPSD, although some bioinformatics skills are still needed to properly install and configure POTION. To bioinformaticians, POTION offers a customizable computational scaffold to perform GSPSD experiments in a controlled and integrated environment. POTION is distributed under GNU General Public License version 3.0 and can be downloaded at http://www.lmb.cnptia.embrapa.br/share/POTION/.

## Implementation

### General overview of POTION workflow

**Fig. 1:**
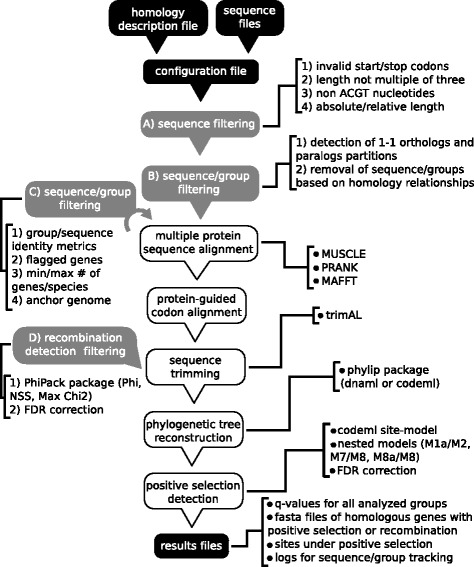
General schema of POTION. *Black boxes* represent user-provided files and final results, *grey boxes* indicate filtering steps, and *white boxes* indicate parallelized steps performed for each valid group of homologs. Filtering steps comprise four sequential conceptual stages (*A*–*D*), each composed of one or more sequential filters (numbered steps). Stage “*A*” comprises four filters for removal of sequence data: (1) absence of valid start and/or stop codons; (2) presence of non-standard nucleotides; (3) length not a multiple of three and (4) lower and upper bounds for sequence length. Stage “*B*” comprises one filter to remove sequences and groups according to homology relationships within groups, allowing users to analyze biologically meaningful gene sets they wish (1-1 orthologs and/or paralogs, for instance). Stage “*C*” comprises four filters for sequences and groups: (1) mean sequence identity of groups or of individual sequences; (2) removal of groups containing any sequence removed in previous steps, allowing users to analyze only high-quality data since the beginning of analysis; (3) removal of groups containing sequence and species count outside user-defined ranges and (4) removal of groups with no sequence from a user-defined anchor genome. Step “*D*” comprises a filter where POTION detects groups with evidence of recombination using three methods (Phi, NSS, Max Chi2), followed by multiple hypothesis correction. After the filtering steps POTION executes the following sequential analyses in parallel for each valid group of homologs: multiple protein sequence alignment using one out of three popular sequence aligners: MUSCLE, MAFFT or PRANK; protein-guided codon alignment; alignment trimming using TrimAl; phylogenetic tree reconstruction using proml and dnaml from phylip; search for positive selection using codeml–site-model analysis using nested models M1a/M2, M7/M8 and M8a/M8, followed by multiple hypothesis correction. POTION parses output files and writes final results files (fasta and flat files) for groups with evidence of recombination and positive selection

POTION is written in Perl language and uses BioPerl modules to handle sequence and alignment data [23]. Our software was developed to explicitly model the procedures commonly performed during GSPSD studies, and can be separated into two main conceptual steps (Fig. 1, grey and white boxes). The first step comprises several sequential sequence and group filtering procedures based on quality and phylogenetic criteria, allowing users to start an analysis with automatically predicted sequence and homology data as input and proceed only with data suitable for downstream analyses. Each filtering procedure is composed of one or more filtering steps that require distinct data to be computed (some of them can be calculated only for aligned sequences, for instance) and are executed when appropriated data types are available (Fig. 1, filtering procedures shown as grey boxes).

All remaining groups after filtering procedures are denominated valid groups and are submitted to the second step of POTION workflow: a parallelized pipeline for positive selection detection comprising five tasks to be computed for each valid group–protein multiple sequence alignment, protein-guided codon alignment, sequence trimming, phylogenetic tree reconstruction and computation of likelihood values for nested codon evolution models (Fig. 1, tasks shown as white boxes). Our software allows users to define several parameters to better model the data under analysis, such as choosing one anchor genome to report results (sequences, IDs and coordinates) in reference to this genome, the genetic code to be used for translation of sequences, additional start/stop codons, or the biological partition of homologs (1-1 orthologs or paralogs) to be evaluated, among many others. A complete description of all parameters is available in “README.txt” file, distributed with POTION.

### First step–data filtering

The first data filtering procedure executed in POTION workflow–Fig. 1, “A) sequence filtering” box–comprises four sequence filters based on the following numeric and quality criteria: (1) absolute and relative sequence length (absolute length filter removes sequences whose lengths fall outside absolute cutoff values; relative length filter removes sequences whose lengths fall outside relative cutoff values in relation to group’s mean/median sequence length); (2) absence of valid start and/or stop codons (user-defined and the ones present in the BioPerl codon tables); (3) presence of non-standard nucleotides; and (4) length not a multiple of three. These filters are intended to remove potential problematic sequences for downstream GSPSD that could inflate false positive error rates through sequence misalignment, such as gene fragments, assembly errors and sequences with extreme length values.

The remaining sequences go through a second filtering procedure–Fig. 1, “B) sequence/group filtering” box–that allows users to select groups and group partitions (subsets of sequences within groups) for downstream analyses through a phylogenetic criterion, namely homology relationships. This filtering step is controlled by the variable “behavior_about_paralogs”, which can be configured to allow users to survey distinct biologically meaningful sets of sequences and groups regarding their homology relationships. At this step users can choose to (1) analyze all groups regardless homology relationships of sequences within them; (2) remove all duplicated genes within a given species (paralogs) in all groups, analyzing only the remaining putative 1-1 orthologs; (3) remove all groups with paralogs, analyzing only “natural” groups of 1-1 orthologs; (4) remove all 1-1 orthologs and analyze all remaining paralogs together regardless the genomes they belong to; and (5) remove all 1-1 orthologs and analyze all remaining paralogs by creating a new subgroup for each genome.

During the individual computation of positive selection for each group other conceptual filtering procedures are available after multiple protein sequence alignment and after sequence trimming. The third conceptual filtering procedure–Fig. 1, “C) sequence/group filtering” box–contains four filtering steps. The first filter step allows POTION to detect and remove highly divergent sequences and/or groups as measured by two metrics calculated by trimAl, each one with cutoff values ranging from 0 to 100. The first metric is used to remove divergent protein sequences, defined as sequences with mean identity (mean of pairwise sequence identity values when comparing a sequence against all others within group) smaller than user-defined cutoff. The second metric aims at removing divergent groups, defined as groups with mean protein sequence identity (mean of pairwise identity values when comparing all sequences within group) smaller than user-defined cutoff. Groups from which sequences were removed due to excessive divergence are realigned after sequence removal with the same parameters before proceeding with analysis.

After this point POTION performs a second quality filter (controlled by the variable “behavior_about_bad_clusters”): the removal of groups which contain any sequence removed during any previous sequence filtering step, allowing users to apply a very stringent filter and analyze only groups that contain high-quality sequence data from the beginning of the analysis. A third quality filter is performed at this point to remove groups that, after the previous filtering steps, contain less or more than the minimum and maximum number of genes/species defined by the user, respectively. POTION computes a fourth optional sequential filtering step where users can define an anchor genome and therefore analyze only groups that, after all the previous sequence filtering steps, contain at least one gene from the anchor genome. POTION will also report all results (sequences, coordinates and IDs) in reference to the anchor genome, generating gene lists that can easily be used in downstream procedures, such as enrichment analysis. At this point users can choose between two anchoring modes: a *strong mode*, where groups without sequences from anchor genomes will be removed, as previously described, and a *weak mode*, where groups that contained sequences from anchor genomes which were removed during any previous filtering step will keep being evaluated. If groups contain more than one sequence from the anchor genome, or if no anchor genome was defined, POTION reports the longest ORF for each genome/group, respectively, or the first ORF to be defined in the homology description file, if there is no length difference between sequences.

After sequence trimming POTION performs a fourth conceptual filtering procedure–Fig. 1, “D) recombination detection filtering” box–to remove groups with evidence of recombination as implemented in PhiPack [24]. PhiPack performs three recombination tests (Phi, NSS and MaxChi2), and POTION can be configured to execute any of these three tests and to require both a minimum number of tests and which individual tests should be significant in order to infer the occurrence of recombination. Since this procedure generates a multiple hypothesis scenario where each group will have an individual *p*-value for each recombination test, users can choose to control the Type 1 errors through the false discovery rate (FDR), configuring POTION to further analyze only genes with corrected *q*-values smaller than user-defined cutoffs for all tests executed. For instance, if users selected (1) the minimum number of positive recombination tests to be two; (2) the algorithm Phi as obligatory to infer recombination detection; and (3) a recombination *q*-value cutoff smaller than 0.1, POTION will remove from downstream analysis all groups where recombination *q*-values are smaller than 0.1 for Phi test (mandatory) and for one out of the two other tests (NSS or MaxChi2). POTION first tries to calculate *q*-values using the qvalue() method as described in [25] and implemented in Statistics:Multtest Perl module, estimating the proportion of true null hypotheses using a bootstrap procedure. If this computation fails for some reason POTION will calculate *q*-values using the BY() method [26].

### Second step–massively parallel search for positive selection

The remaining groups after the filtering steps are considered valid groups and are submitted to the second conceptual step of POTION, which consists on the heavily parallel execution of five sequential tasks for each valid group–multiple protein sequence alignment, protein-guided codon alignment, sequence trimming, phylogenetic tree reconstruction and positive selection detection (Fig. 1, white boxes). Most tasks have execution dependencies, where one or more previously computed data structure must be available to compute the next sequential task for a given group. For this reason each of the five sequential tasks into each group is modeled into POTION as an individual job to be computed when all prerequisites for that job are fulfilled. Also, the computation of each codon evolution model is an independent job in POTION workflow. POTION makes use of computers with multiple processors and implements parallelization using the fork() function implemented in Perl. In order to further increase POTION’s performance we also implemented a line schema for the distinct codeml models where the computation of models prioritizes the order of models to be M8, M8a, M7, M2 and M1a when allocating a new codeml job to a free processor, therefore assuring that the most time consuming models start first during an analysis and minimizing the odds of starting time-consuming models later on.

Users can select some parameters to modify third-party software behavior for each of the five conceptual steps, most of them related to critical speed or quality issues. Ultimately experienced users can edit system calls for each third-party software they wish, or even include other software as needed to tailor the behavior of POTION to better fit their needs. POTION currently supports several established software for the computational steps needed for GSPSD. For multiple sequence protein alignment users can choose one out of three popular multiple sequence aligners: MUSCLE [27], PRANK [28] and MAFFT [29]. A comparative study demonstrated PRANK outperforms MUSCLE for positive selection detection, suggesting PRANK may be the best choice to perform GSPSD [18]. POTION executes MUSCLE with default parameters; PRANK with the flags “-twice” (to run the analysis twice for each group) and “-F” (to correctly penalize the gaps); and MAFFT with “–auto” flag for auto-configuration. Codon alignments are produced by an internal POTION subroutine using protein multiple sequence alignment as a guide.

For alignment trimming POTION currently supports trimAl [30], which can be modified through a single argument: users can supply a numeric argument between 0 and 100 that POTION will use as a lower cutoff for the maximum identity allowed for a given protein column alignment; alternatively users can use strings “strict” or “strictplus” as a parameter, which comprises two stringent filters that take into account column neighborhood for trimming and are recommended by trimAl developers for better reliability. trimAl also computes several identity metrics for each group, which are used to remove sequences and groups with excessive sequence divergence (Fig. 1, third filter procedure). The next step is the phylogenetic analysis, which can be done with the trimmed protein or DNA sequence using the established proml/dnaml programs from the phylip package, respectively [31]. At this point users can select the number of bootstraps for reconstruction of consensus trees and “fast” and “slow” methods implemented in both software.

Lastly, the trimmed codon alignment and the phylogenetic tree files obtained for a given group are used as input files for the codeml program to detect signs of positive selection using site-model analysis [4]. POTION currently supports three popular nested models implemented in codeml (M1a/M2; M7/M8 and/or M8a/M8), and future versions will include other models and branch- and branch-site analyses. The search for positive selection in codeml is done by comparing the log-likelihood values of codon evolution models that do not allow sites with positive selection (M1a, M7, M8a) to the values of the more general nested models that also allow for site classes with positive selection occurrence (M2, M8 and M8, respectively). The *p*-values are calculated as 2Δℓ (twice the difference in likelihood of the two nested models evaluated) based on the *χ*^2^ distribution with 2 ° of freedom for nested models M1a/M2 and M7/M8 and 1 ° of freedom for nested models M8a/M8. Similarly to the recombination analysis, this step is also a multiple hypothesis testing scenario, and therefore POTION also reports corrected *q*-values from the list of *p*-values obtained for all groups evaluated using a given nested model pair (*q*-values are calculated as explained for recombination detection).

## Results and discussion

### Classification accuracy benchmarking

We evaluated POTION in terms of sensitivity, specificity and F-measure (the weighted harmonic mean of precision and recall) by using it to classify a group of high-confidence curated *T. brucei* lineage-specific paralogs previously surveyed for positive selection, henceforth referred to as the TRYP database [15]. This dataset contains 171 genes divided into 40 groups of paralogs, with 23 and 17 groups with and without evidence of positive Darwinian selection, respectively (Additional file 1). TRYP dataset fulfills several criteria to be used as a gold-standard source of homologous genes to evaluate the POTION algorithm as a whole due to the following: (1) it was generated by specialists in trypanosomatid genomics and is expected to represent true, curated groups of homologous genes [32]; (2) all the sequence files are readily available and all groups of homologs are precisely defined; (3) the study evaluated site-model searches for positive selection in both M1a/2 and M7/8 nested codon models; and (4) the authors performed multiple hypothesis correction and reported corrected *q*-values (significance threshold: *q*-value <0.05).

We configured POTION to mimic the original study as much as possible by using the same software versions and parameters, when possible. Specifically we used: (1) MUSCLE (version 3.8.31) to perform multiple protein alignments; (2) trimAl (version 1.2rev59) to filter out alignment columns with more than 50 % gaps; (3) dnaml (phylip version 3.69) for phylogenetic analysis with 100 bootstraps and in fast mode; (4) the nested models M1a/M2 and M7/M8 of codeml (version 3.15) for positive selection detection (*q*-value cutoff <0.05); (5) *T. brucei* genome as anchor; and 6) paralogs-only analysis mode.

When evaluating the TRYP dataset we found 24 groups of homologs with significant evidence of positive selection for both nested codon models, 22 of which were also described as having been positively selected in the original study. POTION misclassified two cases of exclusively purifying selection, identifying evidence of positive selection in two of the 17 groups where previous expert analyses suggests no occurrence of positive selection. POTION achieved values of 0.92, 0.96 and 0.94 for precision, recall and F-measure, respectively ([Additional file 1] contains a table with individual results for each group).

### A case study–*Mycobacterium tuberculosis*

To illustrate how POTION can be used to analyze real genome-scale data towards the identification of reliable positive selection, we used the genomes of 19 *M. tuberculosis* strains as a case study (MYC dataset, [Additional file 2]). Also, to exemplify how the sequential quality filters implemented in POTION could be used to remove noisy data we executed our software using two configuration sets, one with quality filters turned on to remove low-quality data (FILTER experiment) and another without any filtering step based on quality criteria (NOFILTER experiment), and evaluated the results produced by both configuration sets in comparison with similar published GSPSD studies in pathogenic bacteria.

The FILTER experiment was carried out with the following configuration: (1) removal of sequences flagged in any quality filter (absence of valid start/stop codons, ambiguous nucleotides, length not multiple of three) to remove spurious sequence data; (2) removal of sequences whose length falls a) outside the minimum and maximum range of 150 and 100,000 nucleotides (absolute length filter), respectively or b) the range of 20 % the median length of sequences within group (relative length filter), to remove putative gene fragments or other highly divergent sequences in terms of length; (3) removal of any genes from lineages with evidence of paralogy, to analyze only predicted 1-1 orthologs; (4) removal of groups containing sequences identical at the nucleotide level, to avoid spurious computation in non-informative groups; (5) removal of sequences or groups with mean identity lower than 70 %, in order to eliminate highly divergent sequences and groups in terms of similarity; (6) removal of groups that contain less than four genes/genomes after all previous filtering steps; (7) use of the *M. tuberculosis* H37Rv as anchor genome (strong mode); (8) removal of groups with recombination evidence in at least two out of the three metrics implemented in the PhiPack package (version 1.0) (Phi, NSS and Max Chi2, Phi mandatory, *q*-value <0.1 as used in [33]), to avoid possible false-positives in recombination tests; and (9) removal of poor aligning regions using trimAl on “strict” mode. The parameters of NOFILTER experiment are identical to the FILTER configuration, except that all filters that evaluate the quality of sequences, groups, and alignment columns were turned off (filtering steps 1, 2, 5 and 9). Both datasets were analyzed using a multi-core computer with POTION configured to use 90 processors.

We used the scripts distributed with POTION to download and parse the GenBank files corresponding to the genomes of the 19 strains of *M. tuberculosis* and to obtain the predicted coding DNA sequences (CDS) and proteomes for each strain. The predicted proteomes, totalizing 73,933 predicted proteins, were used as input for the OrthoMCL software with default parameters, which predicted 70,445 gene products to belong to one of the 4432 predicted groups of homologs with two or more sequences. POTION was executed using the OrthoMCL main results file that describes the predicted homology relationships and the MYC CDS data as input (data files distributed with POTION).

A total of 7833 individual genes were removed during gene filtering steps in FILTER experiment due to several quality issues, with a median value of 299 genes removed per genome ([Additional file 2] contains the detailed results of genes removed in each *M. tuberculosis* strain). As expected, filters designed to detect extreme values of sequence data distribution in terms of similarity or length, or to remove genes with evidence of paralogy, a fair common evolutionary event, removed genes from the vast majority of MYC genomes. On the other hand, filters related to specific error types in sequence data, such as absence of valid start/stop codons or length not multiple of three, removed genes only from a few genomes. Still, these filters were able to remove hundreds of sequences that could potentially inflate false-positive rates of positive selection detection, such as potential gene fragments and truncated sequences. These filters also detected the highest error count amongst all genomes: *M. tuberculosis* str. Haarlem/NITR202 contains 2878 genes–approximately 90 % of all genes from this strain found in homologous groups–composed of non-standard nucleotides, even though this genomic sequence record is deposited in NCBI as a complete genome, a sequence status commonly related to high-quality data. POTION also removed 1338 groups of homologues in FILTER experiment. A total of 44 groups were removed due to gene/species count lower than cutoff, 575 groups were removed due to absence of a gene from the anchor genome, 717 because they are 100 % identical at nucleotide level, and two because mean group identity was smaller than cutoff. For recombination detection we used the same *q*-value cutoff used by [33], since it appears to achieve an equilibrium between removing true examples of recombination without removing groups likely to be cases of positive selection. No recombination was observed for MYC dataset, even when we used a less stringent *q*-value cutoff (20 %, data not shown). This is coherent with *M. tuberculosis* lifestyle, since it lives mostly in an isolated environment, and is generally believed to be a highly clonal species with a low recombination rate [34].

After the filtering steps POTION selected 3108 and 3624 valid groups of 1-1 orthologs in FILTER and NOFILTER experiments, respectively ([Additional file 3] contains the final results for both experiments produced by POTION). The groups from both experiments were processed with the same pipeline for positive selection, with the exception of alignment trimming using trimAl (version 1.2rev59), only executed in FILTER experiment. We used PRANK (version v.120716) for multiple protein sequence alignment, phylogenetic tree reconstruction using proml (phylip version 3.69) (fast mode, 100 bootstraps) and positive selection detection using codeml (PAML version 4.8) (nested models M1a/M2 and M7/M8, *q*-value <0.05). Even though the tests implemented in codeml and used by POTION to detect positive selection are conservative [35], we decided to use an FDR of 5 % (more stringent than the one used by [33], for instance) to account for the fact we are working with population data, which increases false positive rate [36].

POTION detected 66 and 1218 groups of homologous genes in FILTER and NOFILTER experiments, respectively, where both M2 and M8 models fitted the data significantly better than the simpler nested models, corresponding to 2.1 and 33.6 % of the valid groups of homologous genes evaluated in each experiment (Additional file 4 contains alignment data for FILTER experiment for reproducibility). The computation times for FILTER and NOFILTER dataset were approximately 25 and 50 h, respectively, in multiprocessor mode. Both datasets were computed approximately 60× faster than it would have taken if executed in a single processor. A more detailed analysis of POTION’s behavior when executed in parallel mode can be found in the “Parallelization benchmarking” section. The FILTER dataset generated 5 Gigabytes of raw data after analysis and used approximately a maximum of 2 Gigabytes of RAM.

When analyzing a group of genomes never surveyed for positive selection, metrics such as the ones used in the TRYP experiment to objectively evaluate classification performance in terms of sensibility, specificity and F-measure cannot be applied due to the absence of a “golden truth” reference. For this reason we used the percent of groups of homologous genes under positive selection in studies where authors performed site-based GSPSD in genomes of pathogenic bacteria as a proxy metric for positive selection detection quality. We made this analysis based on the premise that the vast majority of genes are expected to be evolving under stringent purifying selection, and only a minority of adaptive genes will be evolving at accelerated rates. We selected four of such studies that cover a wide range of bacterial lineages and of computational methods to compute positive selection and of filters to remove noisy data: *Listeria monocytogenes* [37]; *Escherichia coli* [10]; *Streptococcus* [38]; and *Actinobacillus pleuropneumoniae* [33].

The *L. monocytogenes* study used TribeMCL [39] for homology inference, ClustalW [40] for protein alignment, a single super-tree using all 1-1 orthologs, and implemented filters to remove sequences with duplications within genomes (paralogs), frameshift mutations or premature stop codons and groups with evidence of recombination. The *E. coli* study used an *in-house* algorithm for homology inference, ClustalW for protein alignment, a super-tree computed using all groups evaluated and implemented filters to remove sequences with low mean similarity, sequences with frameshift mutations, sequences with paralogs and groups with evidence of recombination. The *Streptococcus* study used BlastClust [41] for homology inference, t-coffee [42] for protein alignment, a super-tree from 1-1 orthologs using BIONJ [43] and implemented filters to remove sequences smaller than 100 nucleotides, sequences with frameshift mutations or groups with evidence of recombination. The *A. pleuropneumoniae* study used BlastClust [41] for homology inference, t-coffee for sequence alignment, PhyML [44] for phylogenetic tree reconstruction, and filters to remove sequences with frameshift mutations or smaller than 150 nucleotides and groups with sequences smaller than 80 % of maximum sequence length within group, groups with presence of paralogs or groups with less than four sequences.

As expected, we observed only a small fraction of groups of genes under positive selection in the four studies previously described: *L. monocytogenes*–1.6 % (36 out of 2267); *E. coli*–0.7 % (23 out of 3505); *Streptococcus*–7.9 % (136 out of 1730); and *A. pleuropneumoniae*–3.6 % (57 out of 1587). The percentage of groups of genes under positive selection detected in the FILTER experiment (2.1 %) was much closer to the values observed in these four studies, whereas the NOFILTER experiment (33.6 %) contains a suspiciously higher fraction of genes under positive selection. It is worth mentioning that, although widely used by the scientific community, the results we present for the MYC dataset, as well as for the other four GSPSD studies of pathogenic bacteria, were obtained from population data. In this scenario a considerable number of mutations are not fixed, so instead of estimating dN/dS (ratio of substitution rates), POTION (and the other studies) are estimating piN/piS (ratio of polymorphism rates). Since codeml models were developed assuming somewhat distant lineages with fixed mutations, it is likely that all these results contain a higher rate of false positives [36]. To minimize this issue we used a *q*-value cutoff of 5 % for this experiment, even though similar studies usually choose a much less stringent cutoff [33].

Groups of homologous genes evolving under positive selection in pathogenic bacteria are commonly related to pathogenic lifestyle. Based on this knowledge we performed a literature review to further characterize the 66 groups of homologous genes under positive selection detected in the FILTER experiment and evaluate if POTION selected biologically meaningful groups of homologs under positive selection ([Additional file 5] is a table with annotation information for each of the 66 groups). Several of the groups detected are involved in host-pathogen interactions, such as *otsB2*, a putative peroxidase possibly acting in detoxification reactions [45], *NarJ*, a subunit of a putative respiratory nitrate reductase essential for *M. tuberculosis* maintenance in specific host tissues [46], and *uvrD1*, a DNA helicase known to decrease bacterial pathogenicity when deleted [47].

A considerable number of the genes with roles in host-parasite interaction code for membrane-associated proteins physically located at the host-parasite molecular interface. We found 16 groups of homologous genes under positive selection to belong to the PE/PPE protein families, known virulence factors involved in evasion of the host immune response via antigenic variation and corresponding to up to 10 % of the coding regions of *M. tuberculosis* [48–51]. In fact, a previous study investigated for signs of positive selection in three *M. tuberculosis* genomes and found 12 genes under positive selection, of which six comprised PE/PPE groups [52].

Other membrane-associated gene products under positive selection detected in the FILTER experiment are: (1) locus Rv1635c, which codes for a putative mannosyltransferase predicted to be involved in the biosynthesis of lipoarabinomannan, a glycolipid that plays a major role in host immune system activation and modulation [53]; (2) gene *ddlA*, which codes for a D-alanine-D-alanine ligase involved in peptidoglycan biosynthesis; (3) genes *LpqG* and *LpqM*, coding for two lipoproteins involved in membrane and cell wall processes; and (4) gene *MycP1*, that codes for a membrane-associated serine protease that is a major posttranscriptional regulator of ESX-1, a type VII secretion system used by *M. tuberculosis* to deliver virulence factors into host cells [54]. POTION also detected several genes linked to intermediate metabolism and information pathways to be under positive selection, a phenomenon already observed in other GSPSD studies in parasitic bacteria [10, 37]. We also found several hypothetical groups of homologs with significant signs of positive selection that comprises interesting candidates for further research.

To demonstrate how the anchor genome feature can be used to easily perform downstream enrichment analyses and obtain a broad overview of the biological processes under positive selection in a specific genome, we used the H37Rv strain as anchor genome when analyzing the MYC dataset. The H37Rv is the reference strain for *M. tuberculosis* [50] and is consequently well annotated to distinct biological ontologies, therefore allowing us to directly survey the list of genes under positive selection obtained as output of POTION analysis to observe the functional landscape of positive selection in this species through enrichment analysis [55].

**Fig. 2:**
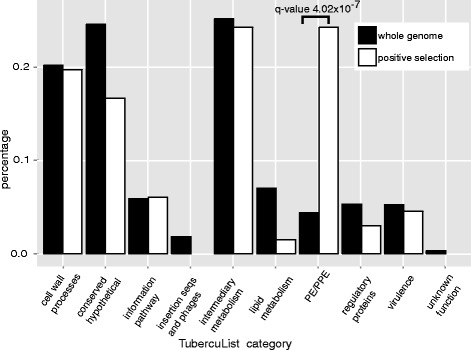
TubercuList categories significantly enriched in positively selected genes in *M. tuberculosis*. The TubercuList category of PE/PPE paralogs is significantly more represented in the list of positively selected genes in H37Rv strain when compared with all coding genes. Count data for positively selected genes was obtained in FILTER experiment and count data for the background frequencies was obtained in the intersection of the list of all valid genes after filtering procedures that are also represented on a given functional category as defined in the TubercuList database [45]

The TubercuList web tool classifies *M. tuberculosis* H37Rv genes into 11 general categories to reflect the main biological roles of this organism, such as metabolism, cell wall processes, virulence factors and pathogenesis [45]. We computed the counts of genes that belong to one out of ten TubercuList categories that contain CDS data (all categories except “stable RNAs”) for both the list of positively selected genes in the FILTER experiment (66 genes) and for all the 3108 groups of valid genes from FILTER experiment (3093 genes). We found the category of PE/PPE paralogs to be significantly enriched (*q*-value <0.05) in the list of positively selected genes ([Additional file 6] and Fig. 2, fisher’s exact test, Bonferroni correction, *q*-value of 4.02 × 10^−7^), strongly suggesting these paralogs are indeed key players to *M. tuberculosis* adaptation to parasitic lifestyle.

Another source of biological information readily available for the H37Rv strain genome is the annotation of CDS data to Gene Ontology (GO) terms through the Blast2GO Functional Annotation Resource [56]. We used this annotation information to perform GO enrichment analysis of all 66 genes under positive selection in H37Rv annotated to at least one GO term (57 genes) when compared with all 3108 valid genes from FILTER experiment that are annotated to at least one GO term (2600 genes) using BINGO with default parameters and a cutoff *q*-value <0.05 [55]. We found 41 significantly overrepresented GO categories ([Additional file 7]). The vast majority of terms are directly related to important mechanisms of *Mycobacterium* host-pathogen interactions such as regulation, modulation and modification of the host immune response, membrane lipid metabolism, several cell wall processes, and receptor mediated binding [50].

### Parallelization benchmarking

**Fig. 3:**
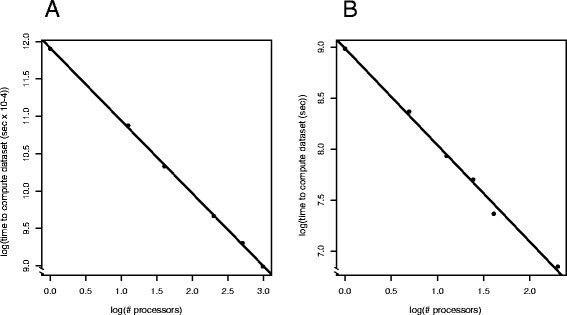
Evaluation of POTION parallelization. **a** Time to compute the first 300 groups of homologs from the MYC dataset while changing the number of processors. **b** Time to compute the TRYP dataset while changing the number of processors. Time decreases in a power-law distribution as the number of processors increases up to the limits of the current algorithm implemented in POTION

The parallelization performance of POTION was evaluated on a multi-core server comprising 96 processors. To evaluate the parallelization schema implemented in POTION we conduced three experiments with the MYC dataset and one with the TRYP dataset. In the first experiment we used a subset comprised of the first 300 groups of homologs predicted by ORTHOMCL (the remaining parameters were the ones used in FILTER experiment) while increasing the number of processors available (Fig. 3a). We observed that the total time taken to compute the whole dataset when increasing the number of processors available for computing appears to follow a power law distribution. We used a log-log plot and fitted a linear model to visualize and estimate the slope of the straight line that best fits the log transformed values (which corresponds to the power law exponent), and found it to be of −0.97. The first increments in the number of processors produce a much larger effect on total computation time than the last increments, suggesting that POTION is reaching the theoretical lower bound of the total time to analyze all groups using the current algorithm.

We monitored POTION execution while varying the amount of groups and processors and found the cause of this phenomenon to be a few groups of homologs for which the time to compute all five tasks (Fig. 1, white boxes) is closer to or greater than the time to compute all other groups with a given amount of processors, causing these groups to be the bottleneck to finish the entire pipeline (see last analysis of this session and [Additional file 8]). We observed that this phenomenon is minimized in situations where the number of processors is much smaller than the number of groups to be computed such that the time to compute bottleneck groups is likely to be smaller than the time to finish computing all other groups, an expected scenario in genome-scale analyses using POTION.

In the second experiment we similarly measured the time to compute the TRYP dataset while increasing the number of processors (Fig. 3b). We also observed that the total time taken to compute the whole dataset to follow a power law distribution when increasing the number of processors (slope of linear regression for log-log plot: −0.95) in a pattern similar to the one observed in MYC dataset (Fig. 3a). POTION was able to compute the TRYP dataset in approximately 129 min with a single processor (7744 s) and took approximately 26 and 15 min (1581 and 942 s, respectively) to compute the same dataset with 5 and 10 processors, respectively, indicating that POTION processed the entire dataset 4.9 and 8.2 times faster with 5 and 10 processors, respectively.

To further address how the time to compute data with POTION scales with the number processors we performed a third experiment where we measured the sum of individual times to compute each individual task when analyzing the entire MYC dataset using the FILTER experiment configuration (3108 groups), as well as the real time taken to compute the same data when allowing POTION to use 90 processors. POTION took approximately 1 day (~25 h, 89,563 s) to compute the entire MYC dataset using 90 processors, and the linear time to compute this entire dataset with a single processor was calculated to be more than 2 months (~1547 h, 5,568,246 s), indicating that POTION processed an entire dataset of genome-scale data 62 times faster when in multi-processor mode.

We performed a fourth experiment to evaluate POTION parallelization schema in order to detect possible fast computational methods to predict groups likely to take a long time to be computed, so those groups could be scheduled first when allocating a free processor, or not analyzed at all. For this purpose we plotted the time to compute the five most CPU intensive tasks (computation of codeml models M1a, M2, M7 and M8 and phylogenetic tree reconstruction) (MYC dataset, NOFILTER experiment, 3108 groups) against several variables intuitively likely to influence the time to compute a task for a given group: (1) number of sequences; (2) alignment length; (3) mean pairwise protein sequence identity; (4) median pairwise protein sequence identity; and (5) standard deviation of pairwise protein sequence identity ([Additional file 8] contains scatterplots and Spearman’s correlation values for these variables).

The profiles observed were very distinct within and between the independent variables evaluated. The number of sequences per group (Additional file 8: Figure S1) appears to be the best overall variable to detect groups likely to spend more time to be computed (correlation values between 0.40 and 0.54). In a lesser extent, the alignment length (Additional file 8: Figure S2) also appears to be a good predictor for computation time, especially for phylogenetic tree reconstruction. In these analyses it was also possible to observe that only a few groups are responsible for the greatest computation times, especially for the two most time-consuming tasks: model M2 (two groups) and model M8 (one group) ([Additional file 8], panels B and D in all figures, respectively. Time-consuming groups are highlighted in red in all graphs). Although these three groups had an elevated number of sequences (18 sequences) with a relative small alignment length (Additional file 8: Figure S2), these variables alone are not sufficient to detect such groups. For instance, the individual time to compute M2 and M8 models for the vast majority of other groups with an equal or greater number of sequences (1515 groups) is at least 300 % smaller than the time for these three extreme cases.

When plotting the time to compute tasks against independent variables that reflect group alignment identity we observed other interesting patterns that could potentially be used to identify groups with higher probability of increased computation time (Additional file 8: Figures S3, S4 and S5). Although no clear correlation was detected (we observed only relatively small negative correlation values), the groups with the highest computation times for models M2 and M8 possess higher mean/median sequence identity and, consequently, smaller standard deviation when compared with the other groups. A possible explanation for the long computation times observed in these groups is that there was not enough time for selection to get rid of mildly deleterious mutations (in the case of codeml models) or that multiple optimal solutions for tree topology are available (in the case phylogenetic tree reconstruction). Taken together, it appears that there may be a pattern to identify groups likely to be time-consuming for specific tasks, such as number of sequences per group in the case of phylogenetic tree reconstruction or groups with a high number of near-identical relatively short sequences in the case of codeml M2 and M8 models. Further studies in this direction will indicate if these metrics could indeed be used to detect such groups and consequently increase POTION’s performance.

Using fast alternatives to compute the results of the most time-consuming steps, such as in phylogenetic tree reconstruction [57, 58] and evaluation of likelihood of distinct codon evolution models [17], in the next versions of POTION are also expected to increase the computational efficiency of our software. Another possibility to further increase the speed of POTION is to use fast methods to detect groups that are likely to be time consuming (such as alignment length for phylogenetic tree reconstruction) in order to allocate more processors to these groups in the case of parallelized third-party software available for a given step.

### Qualitative comparison of POTION with similar software

Due to both the scientific interest and the computational complexity in detecting positive selection in molecular data and on the genome-scale, not surprisingly, there are several software that partially automate some of the computational steps needed for GSPSD. After a thorough literature review we selected six such programs (IDEA [59], JCoDA [60], Datamonkey [61], PhyleasProg [62], Selecton, [63] and PSP [64]) to compare with POTION in order to highlight some of its relative strengths and possible improvements [Additional file 9]. All comparisons were made using the latest available versions of software at the time of publication of this article.

IDEA (Interactive Display for Evolutionary Analysis) is a standalone software that takes one or more user-defined groups of homologs in the form of aligned codons as input, generates nucleotide phylogenetic trees using maximum likelihood (ML) implemented in PhyML [44] or maximum parsimony (MP)/Neighbor-Joining (NJ) methods implemented in phylip. It computes site- and branch-model positive selection using codeml. IDEA contains a Graphical User Interface (GUI) to fully configure the parameters of phylip and PAML and displays results for site-model analyses in an interactive way, including individual sites under positive selection. This software can analyze several groups and tasks within groups in parallel on a local machine or computing grid and offers some extent of user data modeling by allowing the selection of distinct genetic tables.

JCoDA (Java Codon Delimited Alignment) is a second standalone software that takes a single aligned or unaligned user-defined group of homologs as input and performs a codon-delimited alignment using ClustalW [40], followed by phylogenetic tree generation using NJ, MP, or ML as implemented in phylip and positive selection detection using codeml site-model analysis (nested models M1a/M2; M7/M8). JCoDA also provides a GUI to configure software parameters and to display results and also presents some data modeling by allowing the selection of the genetic table to be used.

Other classes of software for automation of positive selection detection are available as web tools. PhyleasProg allows users to select groups of homologs through Ensembl protein IDs from a list of approximately 50 vertebrate species. This software can split groups of mixed homologs into 1-1 orthologs and paralogs and analyze each partition separately. MUSCLE or PRANK are currently available for multiple protein alignment, Gblocks [65] and a home-made tool for sequence trimming, TreeBest for phylogenetic tree reconstruction (unpublished), and evaluates site and branch-site searches using codeml. It is also possible to display positively selected sites on three-dimensional protein structures if structural data is available. PhyleasProg contains some filters implemented, such as the capability to filter groups based on the comparison of alignment lengths before and after sequence trimming, removing groups with very short alignments after this procedure, and splitting of mixed groups in 1-1 orthologs and paralogs, evaluating each gene partition separately.

Datamonkey is also a web tool and corresponds to a front end to some of the unique resources implemented in the HyPhy package [66]. This software analysis starts with an aligned group of homologs and reconstructs phylogenetic relationships using NJ (also accepting user-provided trees) and positive selection detection using some of the methods implemented in HyPhy package. Datamonkey also allows users to select from distinct codon tables and offers positive selection detection methods that take into account the confounding effects of recombination.

Selecton is another web tool that offers automation of positive selection detection. The initial dataset consists on a single group of aligned or unaligned CDS. If users provide only unaligned sequences Selecton performs alignment of translated sequences using ClustalW and produces a phylogenetic tree using NJ. Users are also allowed to submit a Protein Data Bank ID, if available, in order to report results mapped to protein three dimensional structure data. Users can also submit previously aligned codon data and phylogenetic data from other sources. As for positive selection detection Selecton implements five codon evolution models: M5, M7, M8a, M8, and Mechanistic Empirical Combined (MEC) model, the only one that takes into account amino acid replacement rates as well [67]. Users can visualize their results in both primary and tertiary structure data. Selecton also models user data by allowing the selection of distinct genetic tables.

PSP [64] is another example of a web tool developed for GSPSD. Users start an analysis in PSP selecting groups of closely-related prokaryotic genomes and parameters for execution and proceeds with (1) homology relationship inference using OrthoMCL followed by the identification of 1-1 orthologs; (2) protein alignment using MUSCLE or MAFFT; (3) recombination detection using Geneconv [68] and PhiPack; (4) removal of highly divergent sequences using MaxAlign [69]; (5) trimming of alignment columns using in-house scripts; (6) phylogenetic tree reconstruction using MP/NJ (phylip) or ML (CodonPhyML [70]) approaches; and (7) positive selection detection in site- and branch-modes using PAML. PSP contains several functionalities available after positive selection detection, such as visualization of alignments and enrichment analysis of KEGG [71] and GO [72] terms.

Although all of the software evaluated partially automate positive selection detection to some extent, each of them individually contains only a few or even none of the features currently implemented in POTION and, as demonstrated below through a qualitative comparison, none can be used as a general-purpose tool to infer positive selection on user-chosen, genome-scale data in an automated manner ([Additional file 9] contains the qualitative comparison between software).

One of the unique features of POTION is the complete integration with OrthoMCL 1.4 and OrthoXML formats, which allows users to take the output of arguably the most popular homology prediction software and of several databases of predicted homologs and analyze them in a straightforward manner using our software. Regarding filtering procedures, three of the software evaluated do not contain any quality control step (IDEA, JCoDA and Selecton), requiring users to provide curated groups of homologous genes containing only high-quality sequence data. The three remaining contain only one or a few of the following filtering procedures: (1) sequence trimming (PhyleasProg and PSP); (2) recombination detection (Datamonkey and PSP); and (3) removal of divergent sequences in terms of relative length and identity (PSP). In POTION we implemented all the aforementioned plus several other exclusive filters to provide users with a rich set of options to remove noisy data and emulate most filtering criteria commonly used in GSPSD studies (“Implementation” section, data filtering steps).

The lack of most quality filtering steps virtually prohibits the use of these other programs to perform GSPSD using the same initial error-prone data as used by POTION, with the chance of increasing the rate of false detection of positive selection to prohibitive rates [19]. Indeed, as demonstrated in our analysis of MYC dataset (FILTER and NOFILTER experiments), removal of filtering steps when executing POTION can increase positive selection detection to values much higher than the ones observed in other GSPSD, strongly suggesting filtering steps to be a crucial procedure to infer reliable positive selection.

Most of the software we analyzed also offers some features to generalize GSPSD in order to fit the peculiarities of the distinct taxa and gene partitions to be analyzed, such as supporting distinct codon tables (IDEA, JCoDA, Datamonkey, Selecton), searching distinct gene partitions within mixed groups of homologs, such as 1-1 orthologs and paralogs (PhyleasProg), performing recombination detection (Datamonkey and PSP) and using a reference genome to report results (PSP). Nevertheless, only POTION contains all these features implemented in a unique integrated environment. Additionally, POTION also contains exclusive features to further model user data, such as the specification of additional start/stop codons and the removal of entire groups based on phylogenetic and quality criteria.

The automation and generalization achieved in POTION is also unmatched by any of the evaluated software, since all of them either require heavy user intervention at several steps to effectively perform GSPSD or are restricted to a few pre-determined genomes, preventing users to analyze their own data. JCoDA, Datamonkey and Selecton only allow users to analyze a single group per job, rendering them unfeasible to perform any study on a genome-scale in an automated manner. Although IDEA, PhyleasProg and PSP offer the possibility to analyze several groups in parallel, and IDEA can also execute several tasks for the same group in parallel, IDEA requires the sequence data to be previously aligned, and PhyleasProg/PSP do not allow users to analyze most of the sequence data available, being restricted to just a few vertebrate genomes (PhyleasProg) or to strains of closely related prokaryotic genomes (PSP).

Only four programs offer the possibility to align user submitted data: JCoDA and Selecton performs an alignment using ClustalW, an outdated aligner outperformed by PRANK, MAFFT and MUSCLE [18], PhyleasProg and PSP currently supports two out of the three sequence aligners supported by POTION (MUSCLE/PRANK and MUSCLE/MAFFT, respectively). Phylogenetic tree reconstruction is the single step that is present in all software evaluated. Four of them (POTION, IDEA, JCoDA and PSP) use some method from phylip package (IDEA also uses ML from PhyML), Datamonkey and Selection use a NJ algorithm, and PhyleasProg uses TreeBest, an unpublished pipeline. Finally, with the exception of PSP, none of the analyzed software take into account the multiple hypothesis-testing scenarios present during GSPSD.

The qualitative comparison with related software also detected several potential next steps to further develop POTION, such as the support of other third-party programs that could increase POTION’s speed [17, 58]. Also, since POTION implements parallelization only for single machines with multiple processors, IO is likely to be a future lower-bound time bottleneck, since all processes will be reading and writing to the same hard drive during execution. To address this issue we plan to implement parallelization using a message-passing system such as Message Passing Interface (MPI), as implemented in IDEA. Additionally, we also plan to implement other codon substitution models such as EMC (currently implemented in Selecton), which takes into account both mechanistic and empirical data and arguably better fits real sequence data [67]. Also, the possibility to evaluate branch- and branch-site models of positive selection, as seen in several of the software evaluated, will greatly increase the range of biological hypotheses addressed by POTION. Other interesting features can be implemented in sequence trimming procedures where more sophisticated strategies, such as the one implemented to populate the database Selectome [73], could be used to improve the reliability of analysis of more complex genomic data, such as from eukaryotic genomes. Finally, the several GUI available contain interesting features that will be taken into account when developing a future user interface to increase the usability of POTION.

## Conclusions

The search for positive selection in molecular data on a genome-scale is a straightforward option to survey the wealth of taxonomically related genomic data in order to extract biologically meaningful information. POTION aims at providing users an end-to-end pipeline that accepts predicted coding sequence data and homology relationships as input and surveys it in order to offer a reliable detection of positive selection as a final result. We used four complementary strategies to demonstrate the performance and usefulness of our software. The first evaluation strategy consisted of analysis of a highly curated dataset of groups of *T. brucei* paralogs previously surveyed for positive selection (TRYP dataset), used as a gold standard to objectively evaluate the classification efficiency of POTION. Our software achieved a high classification performance, demonstrating that it can effectively distinguish between true positive and true negative cases of Darwinian molecular selection.

To demonstrate how POTION behaves on real, previously unsurveyed data we used our software to perform GSPSD in the genomes of 19 *M. tuberculosis* strains. POTION found several groups of homologous genes with clear roles in host-pathogen biological interactions. Also, in this analysis we demonstrated how some of the unique features implemented in POTION, such as in data filtering and genome anchoring, operate in order to supply users with a rich set of configuration parameters to select partitions of biologically coherent and high-quality sequence data to be further evaluated by POTION. The third analysis strategy consisted of the evaluation of the parallelization schema implemented in POTION, where we demonstrated genome-scale data could be analyzed in a feasible time frame on multi-processor computers executing our software.

Our final analysis consisted of comparing POTION with other software that partially automate the task of GSPSD. We argue that none of the evaluated programs could reliably be used for positive selection detection of genome-scale data, since each of them contain only a few of the functionalities implemented in POTION or, in some cases, none of them at all, such as the capability of analyzing sequence data from virtually any taxa, several of the filters implemented in POTION to remove unreliable data, and correction for multiple hypothesis testing.

We believe POTION is a considerable step towards the automation of an important pipeline in computational genomics, namely automatic detection of positive selection in genome-scale data. It generates a controlled environment allowing single users or small research groups to effectively search for molecular signs of positive selection on their own genomes of interest. Due to its modular nature, advanced users can tailor the POTION scaffold to fulfill their own needs, such as adding new third-party tools that perform analogous tasks. To the best of our knowledge, POTION is the most customizable and general tool to perform positive selection detection available; an end-to-end environment that allows users to construct and check hypotheses regarding the occurrence of site-based evidence of positive selection in genome-scale data within a feasible time frame.

## Availability and requirements

Project name: POTION (POsitive selecTION)

Project home page: http://www.lmb.cnptia.embrapa.br/share/POTION/

Operating system(s): Linux, Unix

Programming language: Perl

Other requirements (and versions for software not mentioned in the main text): Perl packages (BioPerl (1.006901), Cwd (3.47), File::chdir (0.1006), File::copy (2.28), POSIX (1.19), Statistics::Distributions (1.02), Statistics::Multtest (0.13), Tie::File (0.97_02), Try::Tiny (0.11), Data::Dumper (2.131), File::Spec::Functions (3.47), File::Basename (2.78), FindBin (1.50), Capture::Tiny (0.17), Getopt::Long (2.42), PRANK, MUSCLE, MAFFT (6.864b), consense (phylip version 3.69), dnaml, proml, seqboot (phylip version 3.69), PhiPack, TrimAl, codeml.

License: GNU GPL v3

Any restrictions to use by non-academics: no restrictions except the ones stated in GNU GPL v3

## Additional files

Additional file 1:
**Description of TRYP dataset.** Lists the groups of TRYP dataset and their classification as positively selected or not from both the original study and POTION. Additional file 2:
**Description of MYC dataset.** Lists the GenBank IDs of *M. tuberculosis* genomes used in this study (MYC dataset) and the number of genes filtered out by POTION’s filters. Additional file 3:
**Results of FILTER and NOFILTER experiments.** Lists final results produced by POTION when analyzing MYC dataset using FILTER and NOFILTER configuration. Additional file 4:
**Alignment data for MYC dataset.** Compressed text files in .tgz format, untrimmed and trimmed alignment data for MYC dataset as produced by POTION. Data in fasta and phylip format. Additional file 5:
**Genes under positive selection in MYC experiment.** Lists groups of homologs found under positive selection in MYC dataset and their functional annotation from TubercuList. Additional file 6:
**TubercuList categories enrichment analysis of genes under positive selection in** ***M. tuberculosis*** **strain H37Rv.** Lists the percentage of genes annotated to each TubercuList functional category in genes under positive selection (MYC dataset, FILTER experiment) and in all valid genes. Also contains the statistical results for enrichment analysis (Fisher’s exact test, Bonferroni correction, *q*-value <0.05). Additional file 7:
**Gene Ontology enrichment analysis of genes under positive selection in** ***M. tuberculosis*** **strain H37Rv.** Lists Gene Ontology categories significantly enriched in the list of genes under positive selection detected in MYC dataset, FILTER experiment (BINGO, default parameters, *q*-value <0.05). Additional file 8: Figure S1–S5. Correlation between independent variables likely to influence computation time versus time to compute CPU intensive jobs. Contains graphics and Spearman’s correlation values for independent variables likely to influence computation time for a given group (number of sequences, alignment length, mean pairwise sequence identity, median pairwise sequence identity, standard deviation of pairwise identities) versus the time to compute CPU intensive jobs (phylogenetic tree reconstruction / codeml model likelihood calculation). Data obtained from MYC dataset, FILTER experiment. Additional file 9:
**Qualitative comparison of POTION with similar software.** Lists a qualitative comparison of POTION’s features when compared with software that automatizes aspects of genome-scale positive selection detection.

## Abbreviations

CDS: Coding DNA sequence
EMC: Empirical Mechanistic Model
FDR: False Discovery Rate
GUI: Graphical User Interface
GSPSD: Genome-scale positive selection detection
IDEA: Interactive Display for Evolutionary Analysis
JCoDA: Java Codon Delimited Alignment
LRT: Likelihood Ratio Test
ML: Maximum Likelihood
MP: Maximum Parsimony
MPI: Message Passing Interface
NJ: Neighbor-Joining
PSP: Positive Selection for Prokaryotic genomes
