# Supplementary material for: POTION: an end-to-end pipeline for positive Darwinian selection detection in genome-scale data through phylogenetic comparison of protein-coding genes
Source: BMC Genomics. 2015 Aug 1;16(1):567. doi: 10.1186/s12864-015-1765-0 (PMC4521464; doi:10.1186/s12864-015-1765-0)
Supplement: Additional file 8: Figure S1–S5. — Correlation between independent variables likely to influence computation time versus time to compute CPU intensive jobs. Contains graphics and Spearman’s correlation values for independent variables likely to influence computation time for a given group (number of sequences, alignment length, mean pairwise sequence identity, median pairwise sequence identity, standard deviation of pairwise identities) versus the time to compute CPU intensive jobs (phylogenetic tree reconstruction / codeml model likelihood calculation). Data obtained from MYC dataset, FILTER experiment. [file 12864_2015_1765_MOESM8_ESM.pdf]

1 - Number of sequences per group

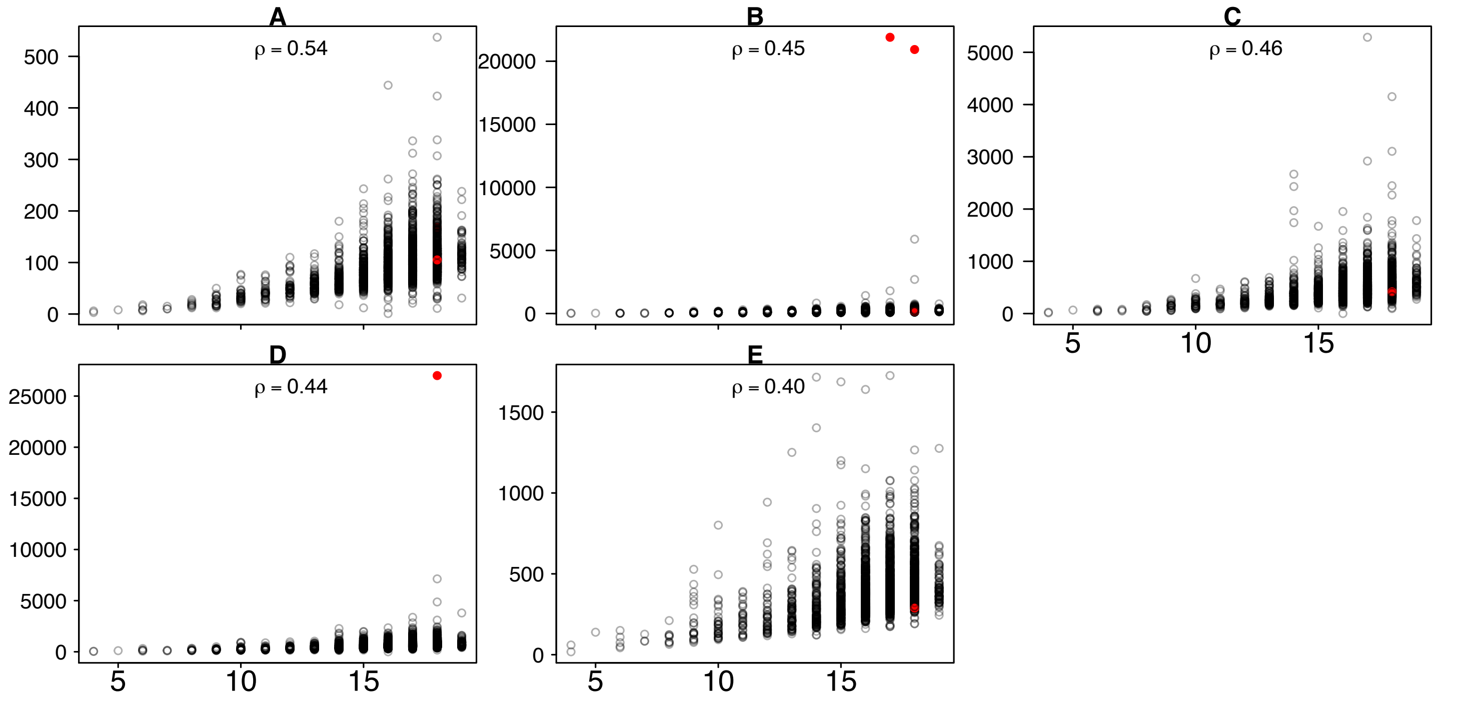

2 - Group alignment length

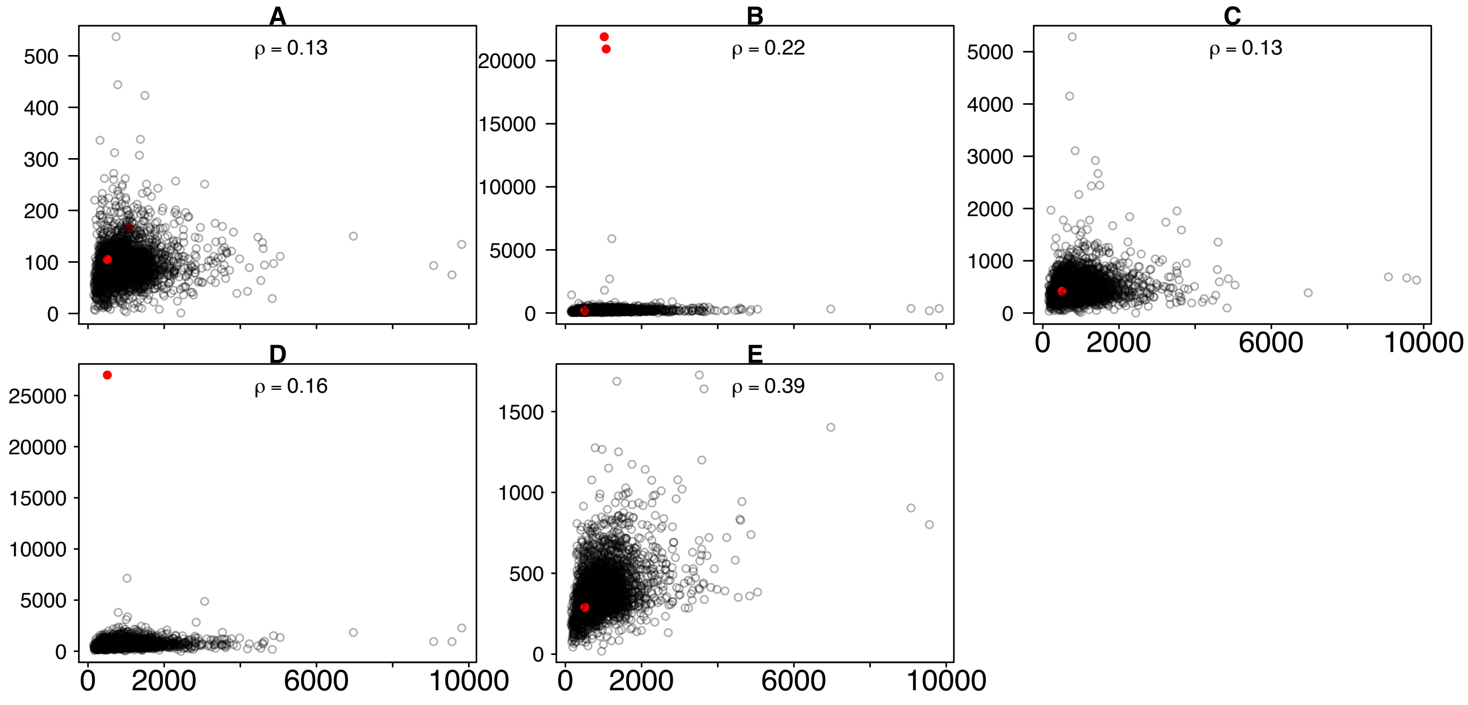

3 - Median group identity for pairwise alignments

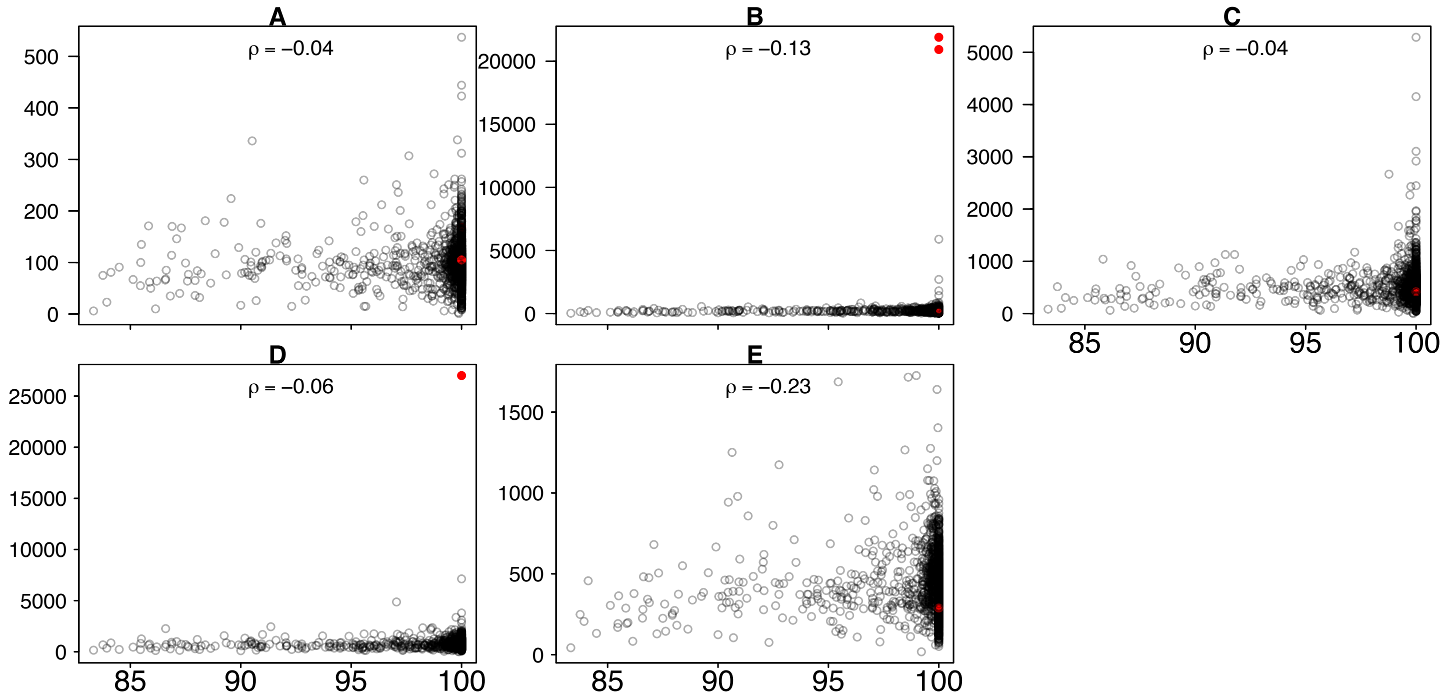

4 - Mean group identity for pairwise alignments

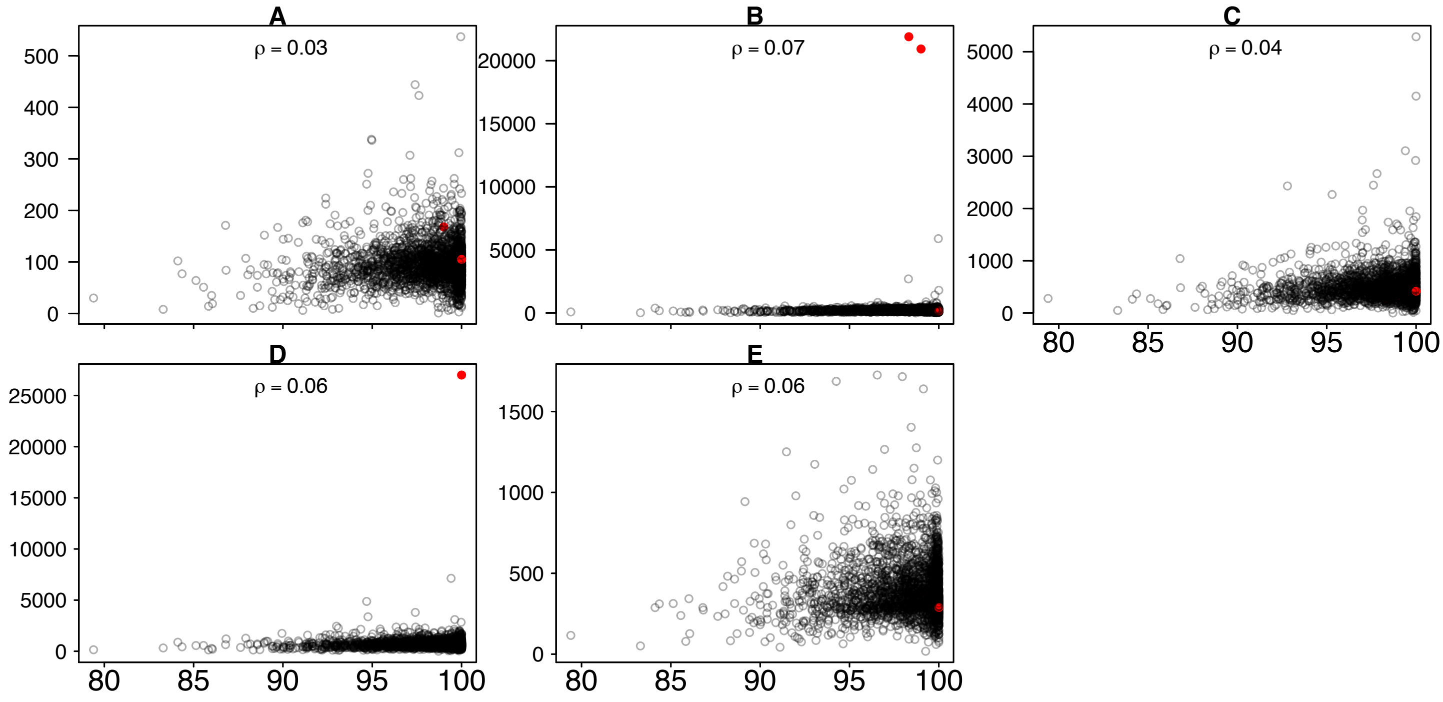

5 - Standard deviation of identities for pairwise alignments

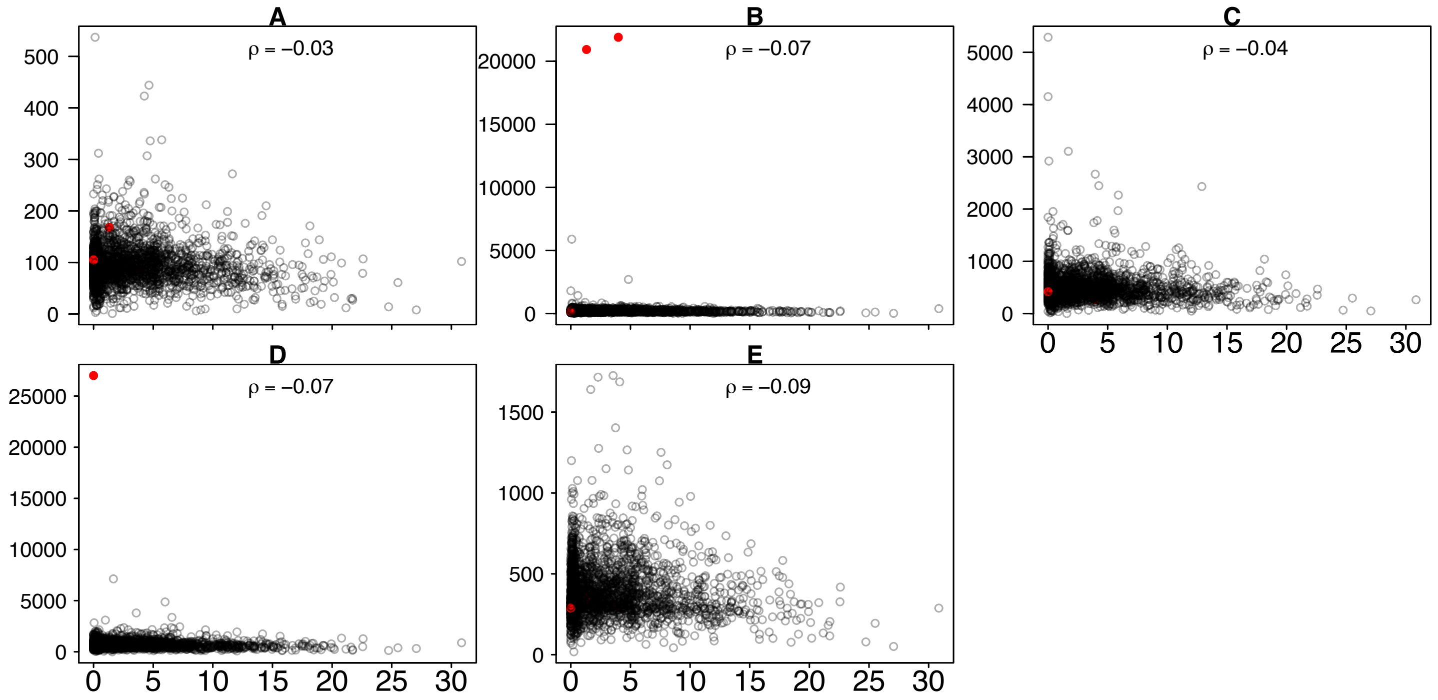

Time to compute a task for a given group of homologous (y-axis) as a function of distinct group properties likely to influence computation time (x axis). A) M1a model; B) M2 model; C) M7 model; D) M8 model; E – phylogenetic tree. Numbers at the top of each graph represent Spearman's correlation coefficient. Time-consuming groups are highlighted in red.
